# Whole-Genome Sequencing of the Novel Pseudomonas Strain TUM22785 Harboring *bla*_PAM-1_, Isolated from a Patient with Urinary Tract Infection in Japan

**DOI:** 10.1128/mra.00148-23

**Published:** 2023-05-30

**Authors:** Shinji Ogihara, Kohji Komori, Shota Miura, Kotaro Aoki, Kageto Yamada, Masakazu Sasaki, Hinako Murakami, Yoshikazu Ishii, Kazuhiro Tateda

**Affiliations:** a Department of Clinical Laboratory, Toho University Omori Medical Center, Ota-ku, Tokyo, Japan; b Department of Microbiology and Infectious Diseases, Toho University School of Medicine, Ota-ku, Tokyo, Japan; Queens College Department of Biology

## Abstract

Pseudomonas species are Gram-negative aerobic bacteria that cause opportunistic infections. Here, we report the whole-genome sequence of the Pseudomonas sp. strain TUM22785, isolated from an outpatient with a urinary tract infection at a medical institution in Japan. This strain harbors a metallo-β-lactamase (MBL) *bla*_PAM-1_ gene.

## ANNOUNCEMENT

We report the genomic characterization of a novel Pseudomonas strain, TUM22785, harboring the metallo-β-lactamase (MBL) gene *bla*_PAM-1_ and isolated via urine culture on 5% sheep blood agar at 35°C for 24 h at the Toho University Omori Medical Center. Cultivation took place under similar conditions. This research was performed according to the Declaration of Helsinki; no specific authorization was issued since the samples were anonymized, and no human data were used. The colony morphology of the strain was similar to that of Pseudomonas aeruginosa. The strain was identified as belonging to the genus Pseudomonas using the MALDI Biotyper Microflex LT/SH system (Bruker Daltonics GmbH, Bremen, Germany), but it was not possible to identify it to the strain level. Antimicrobial susceptibility was assessed following the Clinical and Laboratory Standards Institute guidelines (document M07-A10) (1). The MICs (mg/L) were 64 (ceftazidime), 2 (imipenem), and 4 (meropenem). We monitored carbapenemase production using the modified carbapenem inactivation method (mCIM) and sodium mercaptoacetate (SMA) combination method (2, 3). Both the mCIM and SMA results were positive, confirming phenotypic MBL production in TUM22785.

Bacterial DNA was extracted using the magLEAD and MagDEA Dx SV kits (Precision System Science Co., Ltd., Chiba, Japan), and whole-genome sequencing was then performed on the MiSeq (Illumina, San Diego, CA, USA) and MinION (Oxford Nanopore Technologies, Oxford, UK) platforms. DNA libraries for the MiSeq instrument were prepared using the Illumina DNA prep kit and sequenced using the MiSeq reagent kit v3 (600 cycles; 300-bp paired-end reads) (Illumina). The MinION sequencing libraries were prepared using a rapid barcoding kit and sequenced on an R9.4 flow cell (Oxford Nanopore Technologies). The total numbers of reads, average read lengths, and read *N*_50_ values obtained were, respectively, 1,500,372, 155.95 bp, and 279,010 bp (MiSeq) and 154,469 reads, 17,124 bp, and 21,833 bp (MinION). Base calling and demultiplexing were performed using Guppy v6.3.8. The MiSeq and MinION reads were subjected to quality-based filtering and adapter trimming using Trimmomatic v0.39 (quality score cutoff, 20) (4) and NanoFilt v2.8.0 (quality score cutoff, 6; minimum length, 5,000 bp) (5), respectively. The trimmed reads were hybrid assembled using Unicycler v0.5.0 (6). The contigs were polished thrice using Pilon v1.24 (7). Default parameters were used for all software, unless otherwise specified. Gene annotation was performed using the NCBI Prokaryotic Genome Annotation Pipeline (PGAP) (8).

The assembly genome sequence was obtained as one circular chromosome at an average sequence depth of 377×. TUM22785 had a 66.0 mol% G+C circular chromosome of 7,016,867 bp, with 6,202 CDSs and 4 copies of rRNA operons. Strain TUM22785 did not carry any plasmids. No type strains showed >95% average nucleotide identity against this Pseudomonas strain. We identified an MBL gene (located between positions 2723926 and 2724789 with the locus tag PI990 12645) on the TUM22785 chromosome, and its nucleotide sequences were 100% identical to those of *bla*_PAM-1_ (9).

### Data availability.

The genome sequence of Pseudomonas sp. strain TUM22785 has been deposited at the National Center for Biotechnology Information under BioProject accession number PRJDB14962 and GenBank accession number CP116239. The Sequence Read Archive accession numbers for the raw sequence data are SRX18985619 (MiSeq) and SRX18985620 (MinION).

## References

[1] Clinical and Laboratory Standards Institute. 2015. Methods for dilution antimicrobial susceptibility tests for bacteria that grow aerobically, 10th ed. Clinical and Laboratory Standards Institute, Wayne, PA.

[2] Pierce VM, Simner PJ, Lonsway DR, Roe-Carpenter DE, Johnson JK, Brasso WB, Bobenchik AM, Lockett ZC, Charnot-Katsikas A, Ferraro MJ, Thomson RB, Jr, Jenkins SG, Limbago BM, Das S. 2017. Modified carbapenem inactivation method for phenotypic detection of carbapenemase production among Enterobacteriaceae. J Clin Microbiol 55:2321–2333. doi:10.1128/JCM.00193-17. 28381609 PMC5527410

[3] Arakawa Y, Shibata N, Shibayama K, Kurokawa H, Yagi T, Fujiwara H, Goto M. 2000. Convenient test for screening metallo-beta-lactamase-producing Gram-negative bacteria by using thiol compounds. J Clin Microbiol 38:40–43. doi:10.1128/JCM.38.1.40-43.2000. 10618060 PMC86013

[4] Bolger AM, Lohse M, Usadel B. 2014. Trimmomatic: a flexible trimmer for Illumina sequence data. Bioinformatics 30:2114–2120. doi:10.1093/bioinformatics/btu170. 24695404 PMC4103590

[5] De Coster W, D'Hert S, Schultz DT, Cruts M, Van Broeckhoven C. 2018. NanoPack: visualizing and processing long-read sequencing data. Bioinformatics 34:2666–2669. doi:10.1093/bioinformatics/bty149. 29547981 PMC6061794

[6] Wick RR, Judd LM, Gorrie CL, Holt KE. 2017. Unicycler: resolving bacterial genome assemblies from short and long sequencing reads. PLoS Comput Biol 13:e1005595. doi:10.1371/journal.pcbi.1005595. 28594827 PMC5481147

[7] Walker BJ, Abeel T, Shea T, Priest M, Abouelliel A, Sakthikumar S, Cuomo CA, Zeng Q, Wortman J, Young SK, Earl AM. 2014. Pilon: an integrated tool for comprehensive microbial variant detection and genome assembly improvement. PLoS One 9:e112963. doi:10.1371/journal.pone.0112963. 25409509 PMC4237348

[8] Tatusova T, DiCuccio M, Badretdin A, Chetvernin V, Nawrocki EP, Zaslavsky L, Lomsadze A, Pruitt KD, Borodovsky M, Ostell J. 2016. NCBI Prokaryotic Genome Annotation Pipeline. Nucleic Acids Res 44:6614–6624. doi:10.1093/nar/gkw569. 27342282 PMC5001611

[9] Suzuki M, Suzuki S, Matsui M, Hiraki Y, Kawano F, Shibayama K. 2014. A subclass B3 metallo-β-lactamase found in Pseudomonas alcaligenes. J Antimicrob Chemother 69:1430–1432. doi:10.1093/jac/dkt498. 24356301

